# Supplementary material for: Drebrin controls scar formation and astrocyte reactivity upon traumatic brain injury by regulating membrane trafficking
Source: Nat Commun. 2021 Mar 5;12:1490. doi: 10.1038/s41467-021-21662-x (PMC7935889; doi:10.1038/s41467-021-21662-x)
Supplement: Supplementary file 1 — Supplementary Information [file 41467_2021_21662_MOESM1_ESM.pdf]

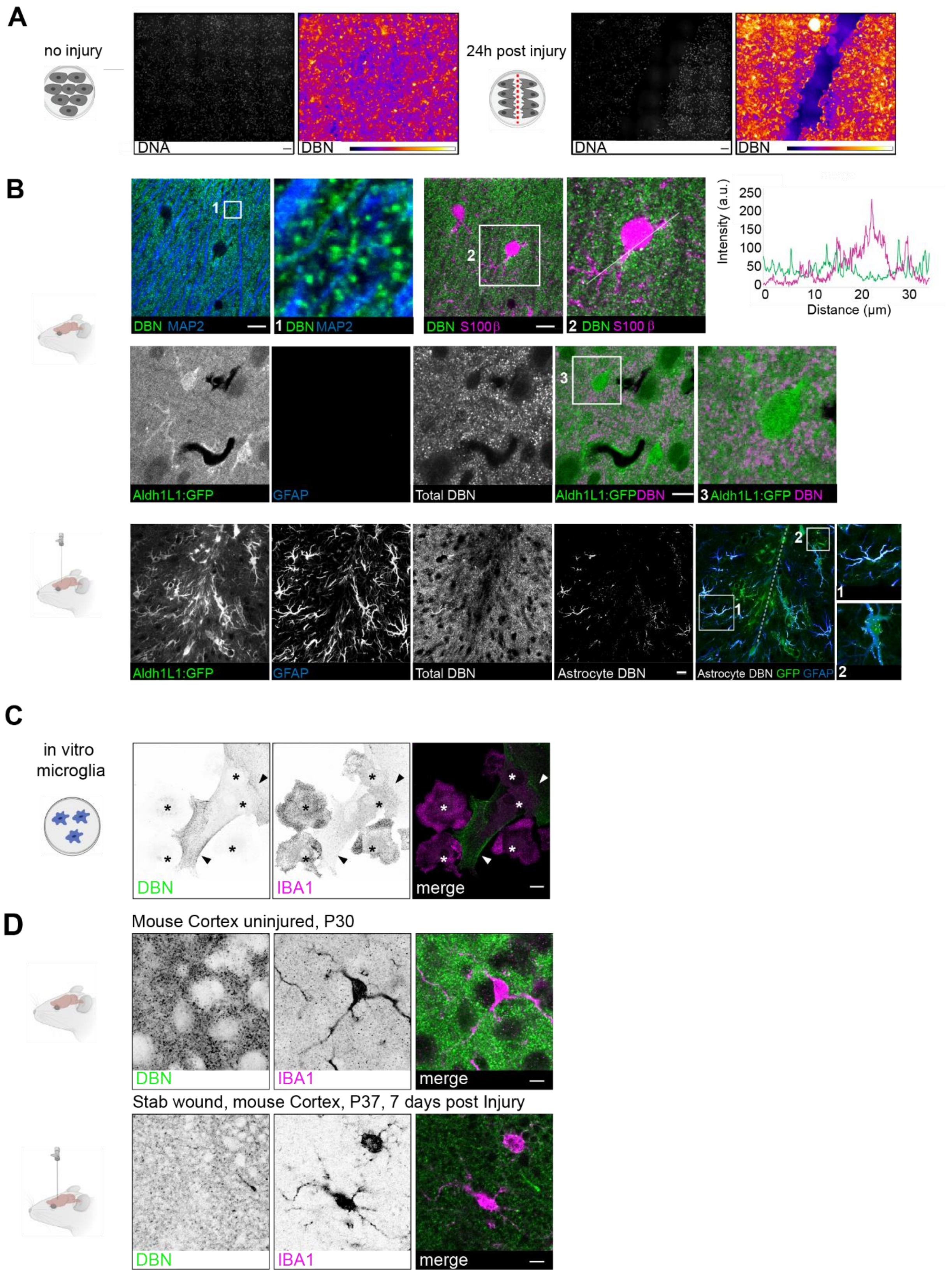

A
no injury
DNA
DBN
24h post injury
DNA
DBN
B
1
DBN MAP2
1 DBN MAP2
2
DBN S100β
2 DBN S100β
Intensity (a.u.)
250
200
150
100
50
0
0
10
20
30
Distance (µm)
Aldh1L1:GFP
GFAP
Total DBN
3
Aldh1L1:GFP DBN
3 Aldh1L1:GFP DBN
Aldh1L1:GFP
GFAP
Total DBN
Astrocyte DBN
2
1
Astrocyte DBN GFP GFAP
1
2
C
in vitro
microglia
DBN
IBA1
merge
D
Mouse Cortex uninjured, P30
DBN
IBA1
merge
Stab wound, mouse Cortex, P37, 7 days post Injury
DBN
IBA1
merge

Supplementary Figure S1: Injury-dependent upregulation of DBN in astrocytes. (A) Widefield mosaic scan of 25 fields of view from confluent astrocyte cultures labeled with anti-DBN, without or 24 h after mechanical injury. Relative DBN fluorescence is displayed as heatmap. Scale bars: 100 µm. Representative image of 3 independent experiments. (B) IHC of uninjured or injured mouse brain. Upper panel shows a strong labeling of DBN (green) around MAP2+ dendrites (blue), and no DBN labeling in S100ß+ astrocytes (magenta) in the uninjured mouse brain. Images are single confocal sections. Representative images of 2 animals. Scale bars: 10 µm. Close up image (1) show that DBN+ structures emerge from MAP2+ dendrites, which identifies them as dendritic spines. Line-scan through an S100ß+ astrocyte in close up image (2) shows little overlap of DBN and S100ß signal. Center panel shows IHC of DBN protein in uninjured BAC Aldh1l1 EGFP mice. Aldh1l1:GFP+ astrocytes (green) are negative for GFAP (blue) and DBN (magenta) in uninjured settings. Bottom panel shows IHC of DBN expression (grey) in BAC Aldh1l1 EGFP mice 7 days post-stab-injury. Astrocyte reactivity at lesion sites was determined by GFAP (blue), whilst astrocytes independent of their reactivity were visualized by GFP expression (green). Magnifications demonstrate astrocytes exhibiting prominent (1) and moderate (2) DBN protein levels. Images are confocal stacks. Representative images of 2 animals. Scale bars: 20 µm. (C) Microglia-enriched cultures were labeled for DBN (green) and the microglia marker IBA1 (magenta). IBA1+ microglia are negative for DBN (asterisks), whilst neighboring reactive astrocytes (arrowheads) are DBN+. Representative images of 3 experiments. Scale bars: 10 µm.(D) IHC of DBN (green) and IBA1 (magenta) in the uninjured cortex of P30 WT mice or 7 days post injury. Representative images of 3 animals. Scale bars: 10 µm. Images in (C) and (D) are single confocal planes. Source data are provided as a Source Data file.

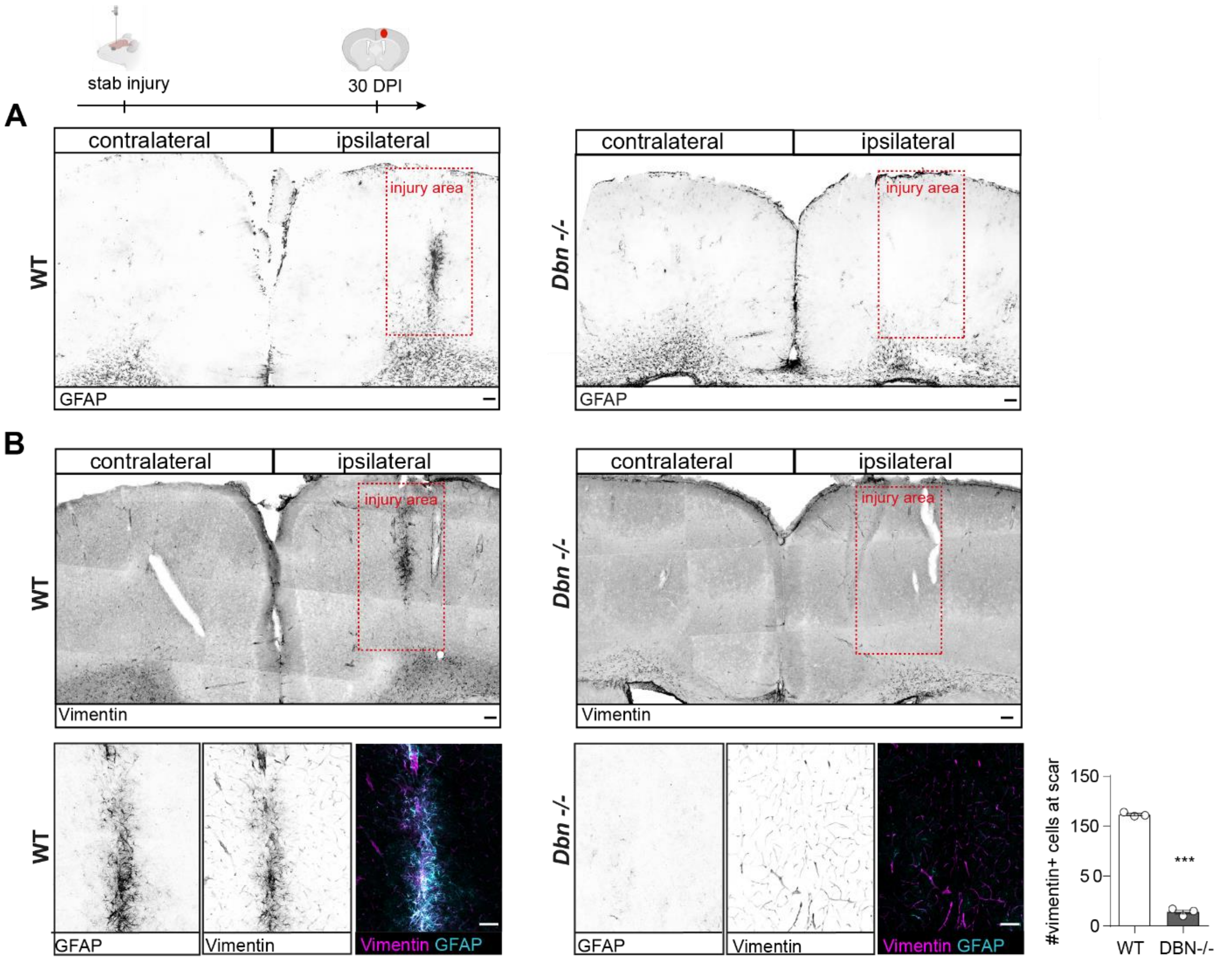

Supplementary Figure S2: Distribution of the reactive astrocyte markers GFAP and vimentin in WT and *Dbn*$^{-/-}$ cortices. (A) Overview images of WT and *Dbn*$^{-/-}$ cortices stained for GFAP 30 days post stab injury. Uninjured cortical tissue show little GFAP immunoreactivity. White matter astrocytes in the adjacent corpus callosum are overall GFAP+. WT but not *Dbn*$^{-/-}$ cortices show GFAP+ reactive astrocytes at stab wounds (dashed box). Representative images of 11 WT and 13 *Dbn*$^{-/-}$ animals. Scale bars: 100 µm. (B) Overview images of WT and *Dbn*$^{-/-}$ cortices stained for vimentin 30 days post stab injury (upper panel). In WT brains, the vimentin antibody labels blood vessels and reactive astrocytes at stab wounds (dashed box). *Dbn*$^{-/-}$ cortices lack vimentin+ astrocytes at injury sites. Scale bars: 100 µm. Bottom panel shows stab injuries labeled for GFAP and vimentin. Reactive WT astrocytes were positive for both GFAP and vimentin. Injury sites in DBN *Dbn*$^{-/-}$ brains exhibit vimentin signals only around blood vessels. Scale bars: 100 µm. Quantification of vimentin+ cells at stab wounds 30 DpI shows means, individual data points and SEM; n=3 animals, *** P= 0.00000219 (Students unpaired t-test, two-sided, t=40.63 df=4). All images are confocal stacks. Source data are provided as a Source Data file.

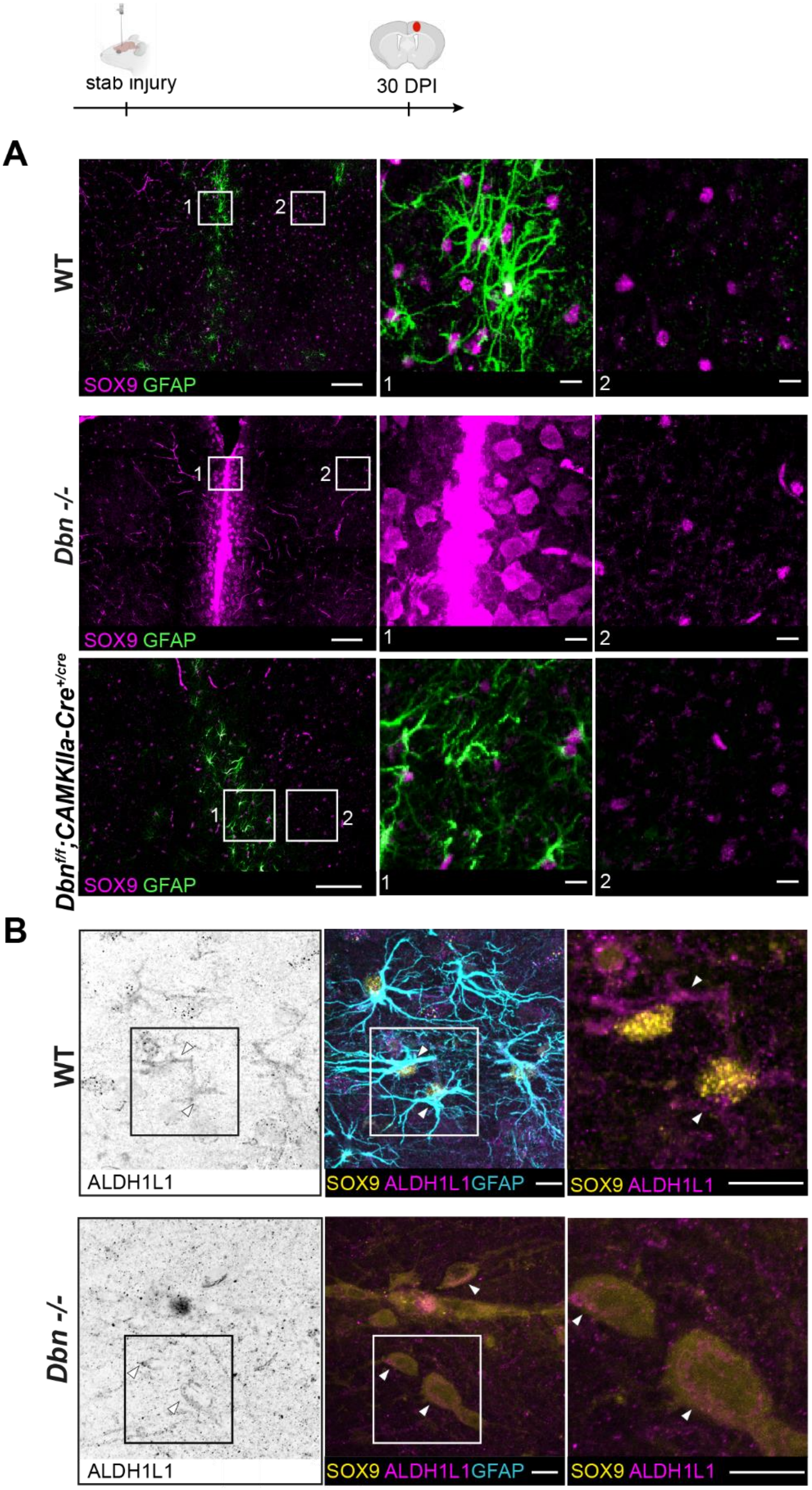

stab injury
30 DPI
A
WT
Dbn -/-
Dbn$^{f/f}$;CAMKIIa-Cre$^{+/cre}$
SOX9 GFAP
1
2
B
ALDH1L1
SOX9 ALDH1L1GFAP
SOX9 ALDH1L1

Supplementary Figure S3: Analyses of SOX9+ cells at stab wounds of WT, *Dbn*$^{-/-}$ and *Dbn*$^{fl/fl}$*; CAMKIIa-Cre*$^{+/cre}$ mice (30 DPI). (A) IHC of stab wounds labeled for SOX9 (magenta) and GFAP (green) in WT (upper panel), *Dbn*$^{-/-}$ (center panel) and *Dbn*$^{fl/fl}$*; CAMKIIa-Cre*$^{+/cre}$ brains (bottom panel). Close up images (1) show lesion sites, while close up images (2) magnify uninjured tissue. Scale bars overviews: 100 µm; Scale bars close up images: 10 µm. Representative images of 3 animals/condition. Images are confocal stacks (B) Triple labeling of ALDH1L1 (magenta), SOX9 (yellow) and GFAP (cyan) in stab wounds of WT and *Dbn*$^{-/-}$ brains (30 DPI). Arrow heads indicate ALDH1L1 signals in cell bodies of SOX9+ cells. Representative images of 3 animals/condition Scale bars: 10 µm. Overviews are confocal stacks, while close ups are single confocal sections. Source data are provided as a Source Data file.

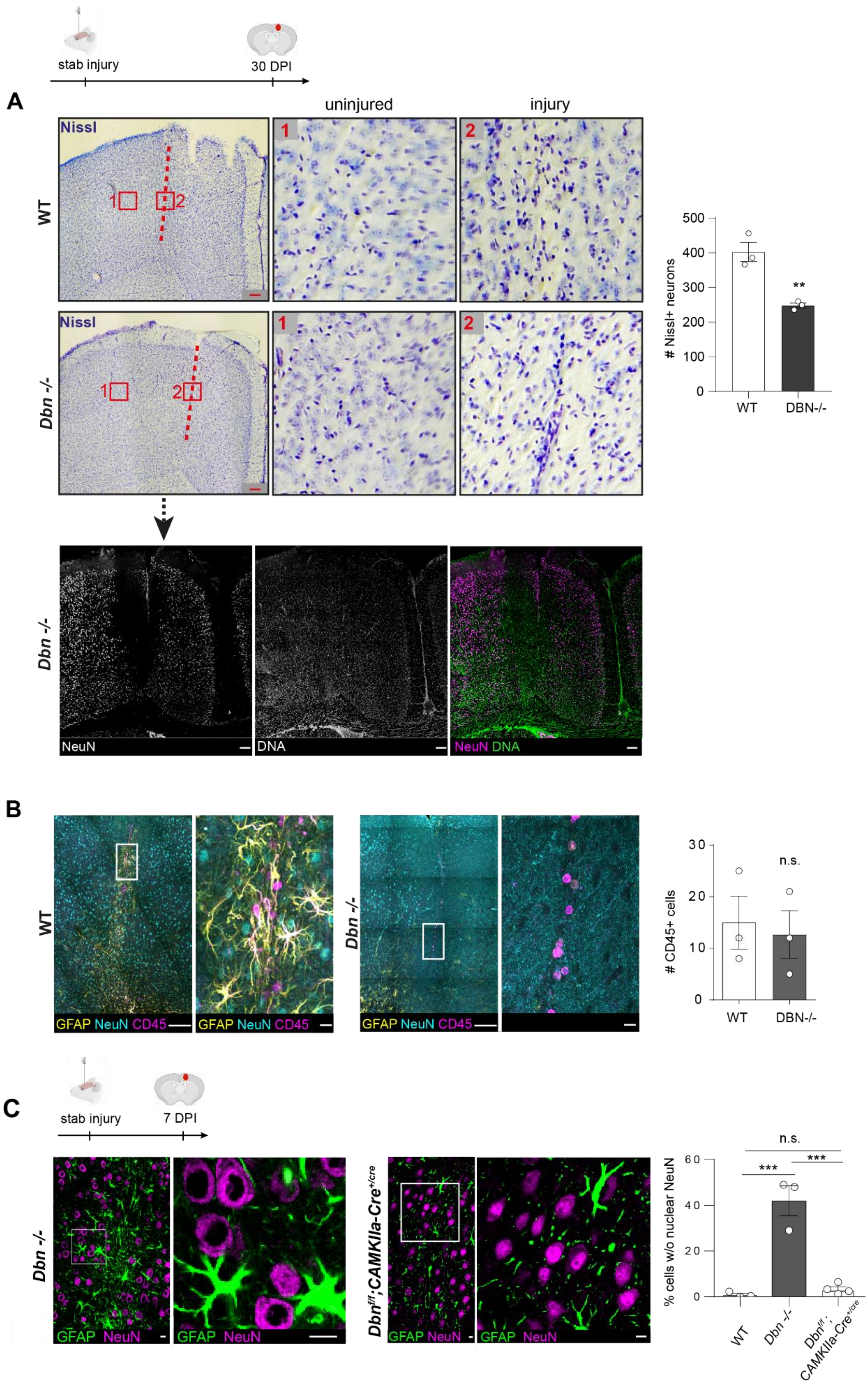

stab injury
30 DPI
A
uninjured
injury
Nissl
1
2
WT
Dbn -/-
Nissl+ neurons
500
400
300
200
100
0
**
WT
DBN-/-
NeuN
DNA
NeuN DNA
B
GFAP NeuN CD45
CD45+ cells
30
20
10
0
n.s.
C
7 DPI
GFAP NeuN
Dbnf/f;CAMKIIa-Cre+/cre
% cells w/o nuclear NeuN
60
40
20
0
***
Dbn -/-

Supplementary Figure S4: Histological and immunohistochemical analyses of stab wounds in WT, *Dbn*$^{-/-}$ and *Dbn*$^{fl/fl}$*; CAMKIIa-Cre*$^{+/cre}$ mice (30 DPI). Nissl labeling of cortices from WT and *Dbn*$^{-/-}$ mice after stab wounding (30 DPI). Close up images (1) show uninjured tissue. Close up images (2) show tissue around stab wounds (dashed lines). Scale bars: 100 µm. Quantification of neurons, characterized by large Nissl+ cell bodies, 300 µm around the lesion sites, displayed as bar graphs showing mean, individual data points and SEM. n=3, ** *P*=0.0055 (Unpaired t-test, two-sided t=5.448, df=4). Lower panel shows the same *Dbn*$^{-/-}$ brain slice as above stained for NeuN (magenta) and DNA (green). (B) WT and *Dbn*$^{-/-}$ brains after stab wound (30 DPI) labeled for GFAP (yellow), NeuN (cyan) and CD45 (yellow). Scale bars: overview: 100 µm, magnification: 10µm. Quantification (means, individual data points and SEM) of CD45+ cells in stab wounded WT and *Dbn*$^{-/-}$ brains. n=3 animals, n.s. *P*=0.7527 (Unpaired t-test, two-sided, t=0.3376, df=4). (C) IHC of GFAP (green) and NeuN (magenta) in *Dbn*$^{fl/fl}$*; CAMKIIa-Cre*$^{+/cre}$ mice (7 DPI). Scale bars: 10 µm. Bar chart shows quantification (means, individual data points and SEM) of neurons without nuclear NeuN at injury sites of *Dbn*$^{fl/fl}$*; CAMKIIa-Cre*$^{+/cre}$ mice (7 DPI) and WT and *Dbn*$^{-/-}$ mice, as shown in Figure 2C. n=3 for WT and *Dbn*$^{-/-}$, 4 for *Dbn*$^{fl/fl}$*; CAMKIIa -Cre*$^{+/cre}$ (One-way ANOVA F=42.89, DFn=2, DFd=7; Tukey's multiple comparisons test: WT vs *Dbn*$^{-/-}$ *** *P*= 0.000214, WT vs. *CAMKIIa-Cre*$^{+/cre}$ n.s. *P*= 0.8631, *CAMKIIa-Cre*$^{+/cre}$ vs *Dbn*$^{-/-}$ *P*= 0.000208). All images are confocal stacks. Source data are provided as a Source Data file.

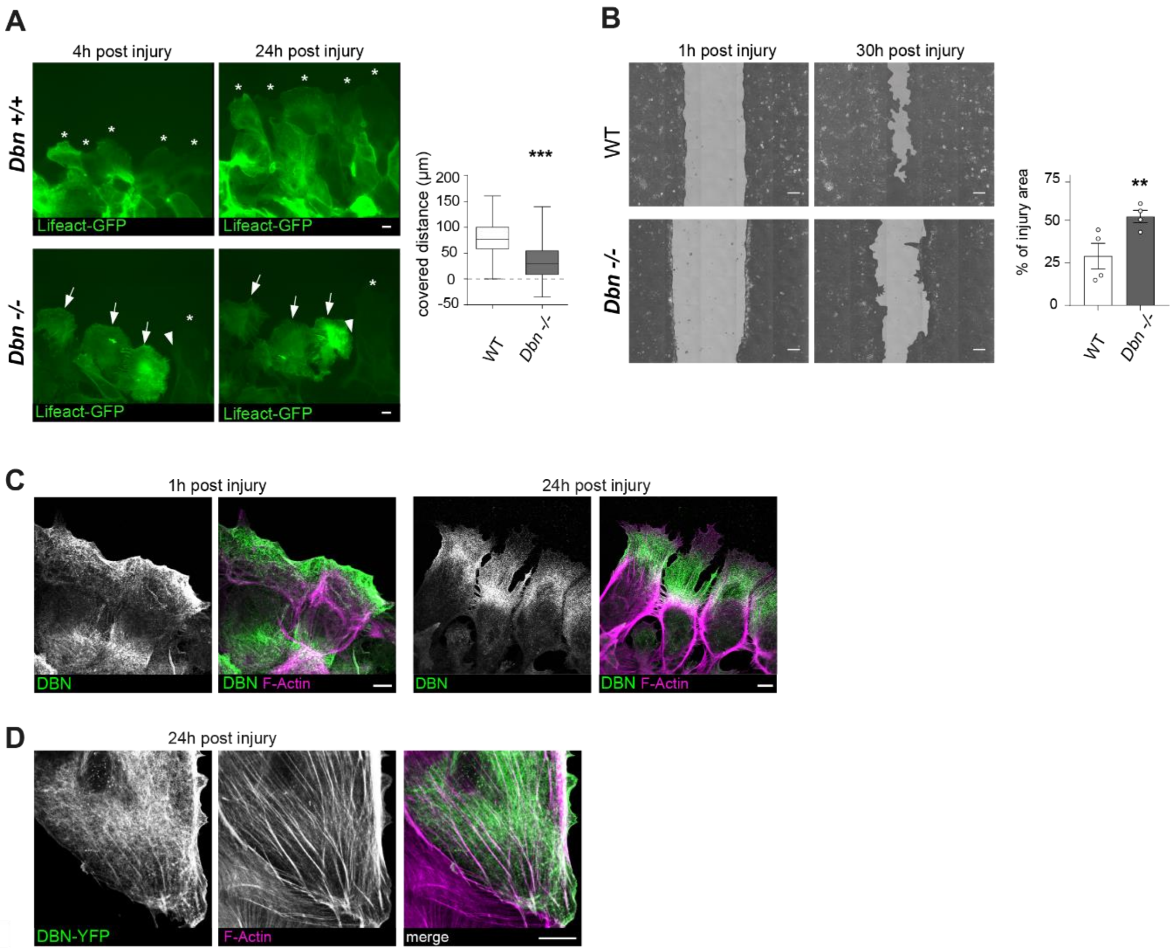

Supplementary Figure S5: DBN-loss perturbs the coordinated outgrowth of cultured astrocytes after scratch injury. (A) WT and *Dbn*$^{-/-}$ astrocytes expressing Lifeact-GFP, at 4 h and 24 h after mechanical injury *in vitro*. Asterisks indicate cells with extending processes in a persistent manner towards injury. Arrows show moving cells with erratic migratory behavior. The arrowhead show retracting astrocytes. Scale bars: 10 µm. Images were acquired by widefield microscopy. Quantification shows box and whisker plots (box extends from 25th to 75th percentiles, central line=median, whiskers comprise all values from minimum to maximum) of distances covered by WT and *Dbn*$^{-/-}$ astrocytes; n=180 cells per group obtained from three independent experiments, *** $P < 0.0001$ (Students unpaired t-test, two-sided, t=13.2 df=358). (B) Live imaging of the overall wound closure of cultured WT and *Dbn*$^{-/-}$ astrocytes after 1 and 30 h. Scale bars: 100 µm. Bar diagram shows quantification of wound size 30 h after injuring the astrocyte monolayers (means, individual data points and SEM); n=4 independent experiments, Two-way repeated measurements ANOVA, F= 7.804, DFn=1, DFd=6; Sidak's multiple comparisons test: ** P=0.0038). (C) DBN and F-Actin labeling in cultured astrocyte during scratch injury. 1h post injury, DBN localizes to the rear and leading edge of injured astrocytes and shows little co-localization with the most prominent actin filaments. 24h after injury, DBN is detected between the leading edge and the actin-rich cell body, showing again little co-localization with typical actin fibers. Representative images of 3 independent experiments. Scale bars: 10µm. (D) Localization of DBN-YFP in vesicular and tubular structures and, partially, on actin fibers. Scale bar: 10µm. Representative images of 3 experiments. Images in (C) and (D) are single confocal sections. Source data are provided as a Source Data file.

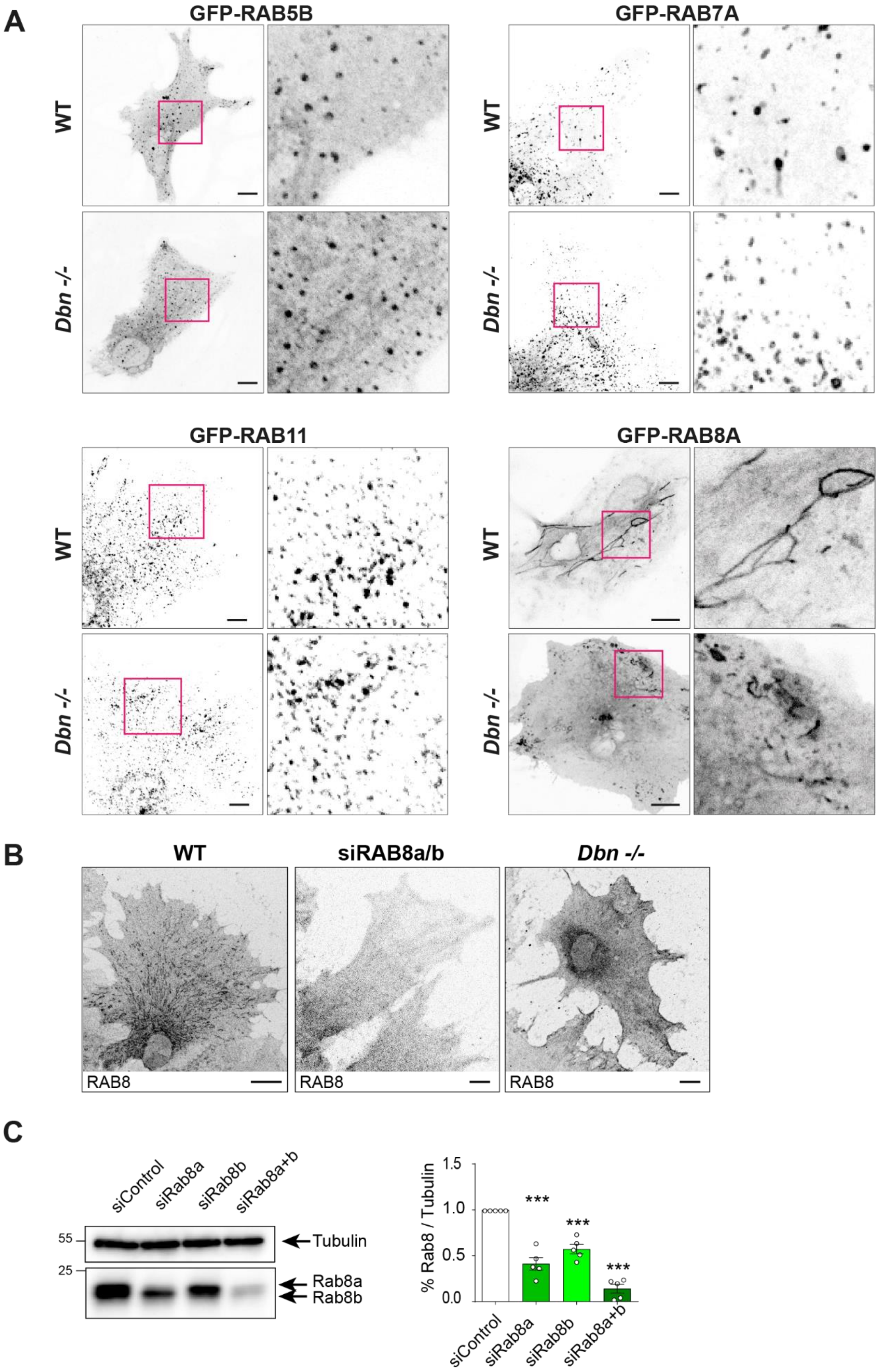
A
GFP-RAB5B
GFP-RAB7A
WT
Dbn -/-
GFP-RAB11
GFP-RAB8A
B
WT
siRAB8a/b
Dbn -/-
RAB8
C
siControl
siRab8a
siRab8b
siRab8a+b
55
25
Tubulin
Rab8a
Rab8b
% Rab8 / Tubulin
1.5
1.0
0.5
0.0
***

Supplementary Figure S6: Localization and specificity RAB-GTPases in astrocytes during injury. (A) Expression of different GFP-tagged RAB GTPases in cultured WT and *Dbn*$^{-/-}$ astrocytes. No major differences between genotypes could be detected for RAB5b, 7A and 11; RAB8A formed tubular structures in WT but not in *Dbn*$^{-/-}$ astrocytes. Representative images of 3 experiments. Scale bars: 10 µm. (B) WT astrocytes, WT astrocytes transfected RAB8A+ siRNA and *Dbn*$^{-/-}$ astrocytes; demonstrating the specificity of the antibody used. Scale bars: 10 µm. Representative images of 3 independent experiments. Image are confocal stacks. (C) Western blot of RAB8 levels using a pan RAB8 antibody in lysates derived from WT astrocytes transfected with control siRNA (siControl), Rab8a specific siRNA (siRab8a) and/or Rab8b specific siRNA (siRab8b). Tubulin immunoreactivity serves as loading control. Rab8 and Tubulin antibody incubations were performed on the same membrane on consecutive days without stripping. Bar diagram shows quantification of RAB8 levels from siRNA transfected astrocytes from corresponding western blots, displaying means, individual data points and SEM (n=5 independent experiments, One way ANOVA F= 55.82 (DFn=3,DFd= 16), Bonferroni's multiple comparisons test ***P<0.001; multiplicity adjusted p-values: siControl vs siRab8a: P= 0.000001199564256; siControl vs siRab8b: P= 0.000065561231107; siControl vs siRab8a+b: P= 0.000000005946593). Source data are provided as a Source Data file.

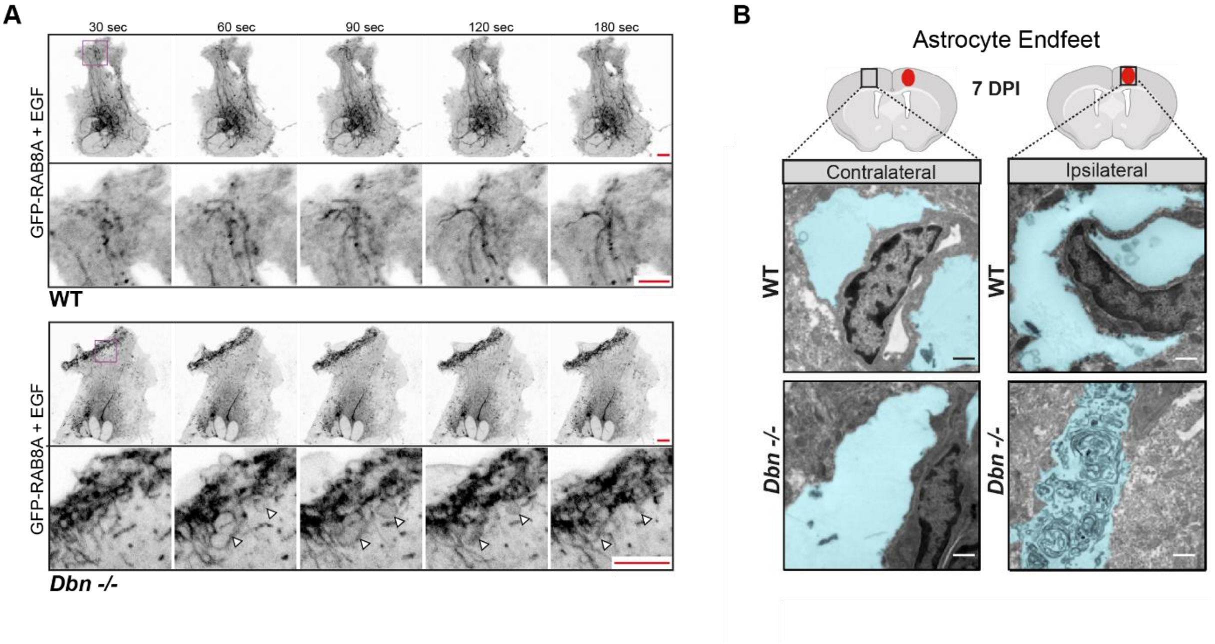

Supplementary Figure S7: DBN-loss results in accumulation of membranes in astrocytes. (A) WT and *Dbn*$^{-/-}$ astrocytes expressing GFP-RAB8A after starvation and brief stimulation with EGF (see Movies 5 and 6). Images are confocal stacks acquired during live imaging. Representative images of 3 experiments. Scale bars: 10 µm. Images are confocal stacks. (B) TEM images showing accumulation of multilamellar bodies in endfeet of astrocytes in *Dbn*$^{-/-}$ brains, but not in endfeet of astrocytes in WT brains after injury *in vivo* 7 DPI. Astrocyte endfeet are shaded in blue. Representative images of 3 animals/condition. Scale bars: 500 nm. Source data are provided as a Source Data file.

Supplementary Table S8 .List of primers.

Name	Sequence 5'-3'	Purpose
pCDF-New MCS-s	CTAGAGCTAGCGCTACCGGTCGCCACCATGGGATGTACAGCGGCCGCG	Cloning
pCDF-New MCS-as	TCGACGCGGCCGCTGTACATCCCATGGTGGCGACCGGTAGCGCTAGCT	Cloning
pGFAP-5	ATAGATATCAACATATCCTGGTGTGGAGTAGGG	Cloning
pGFAP-3	ATAGCTAGCGCGAGCAGCGGAGGTGATGCGTC	Cloning
pCDF-GFP-5	GACCTCCATAGAAGATTCTAGAGCTAGCATGGTGAGCAAGGGCGAGGAGCTGTTC	Cloning
pCDF-RAB8A-3	GTAATCCAGAGGTTGATTGTCGACTCACAGAAGAACACATCGGAAAAAGCTGC	Cloning
Paxillin-BsrGImut-s	GAGGAGGAACACGTGTATAGCTTCCCAAACAAGCAG	Mutagenesis
Paxillin-BsrGImut-as	CTGCTTGTTTGGGAAGCTATACACGTGTTCCTCCTC	Mutagenesis
CAMKII Cre FW wt+cre	GGTTCTCCGTTTGCACTCAGGA	Genotyping
CAMKII Cre B RV cre	CCTGTTGTTCAGCTTGCACCAG	Genotyping
CAMKII C B RV WT	CTGCATGCACGGGACAGCTCT	Genotyping
siRAB8A	GGAAUAAGUGUGAUGUGAA	RNAi
siRAB8B	GAAUGAUCCUGGGUAACAA	RNAi
siControl	AGGUAGUGUAAUCGCCUUGUU	RNAi
