# Supplementary material for: Drebrin controls scar formation and astrocyte reactivity upon traumatic brain injury by regulating membrane trafficking
Source: Nat Commun. 2021 Mar 5;12:1490. doi: 10.1038/s41467-021-21662-x (PMC7935889; doi:10.1038/s41467-021-21662-x)
Supplement: Supplementary file 3 — Description of Additional Supplementary Files [file 41467_2021_21662_MOESM3_ESM.pdf]

Description of Additional Supplementary Files

Title: Supplementary Movie 1

Description: Animated orthogonal projection, corresponding to close up image (1) in Figure S1B, shows Aldh1l1:GFP+ reactive astrocytes (green) counterstained for DBN (magenta) and GFAP (blue) after stab injury (7 DPI).

Title: Supplementary Movie 2

Description: Live imaging of cultured WT (left) and Dbn-/- (right) astrocytes expressing Lifeact-GFP after mechanical injury, corresponding to Supplementary Figure 3. Video acquisition began 4 h and ended 24 h after mechanical injury.

Title: Supplementary Movie 3

Description: Live imaging of cultured WT (left) and Dbn-/- (right) astrocytes expressing GFP-RAB8A in conjunction with the parallel visualization of our automated and quantitative tubule detection algorithm. Video recording started 24 hours after injury. Tubules were recorded for 30 min.

Title: Supplementary Movie 4

Description: Live imaging of originally GFP-RAB8A tubule deprived Dbn-/- astrocytes mostly after adding Cytochalasin D (left), CK-666 (center) and SMIFH2 (right). Video recording started right after adding the reagents. The effects of the inhibitors were followed for 30 min.

Title: Supplementary Movie 5

Description: Time-lapse video of cultured WT (left) and Dbn-/- (right) astrocytes during EGF-stimulation. After 24 h injury, cells were starved for 2h and subsequently treated with EGF. Video recording started immediately after adding EGF. The effect of EGF was followed for 10 min.

Title: Supplementary Movie 6

Description: Magnifications on leading edges of EGFtreated WT (left) and Dbn-/- (right) astrocytes according to Movie 4. Note that long extending tubules are formed in Dbn+/+ astrocytes via smaller particles. EGF treatment leads to membrane accumulation in Dbn-/- astrocytes with short tubules and instable vacuole-like structures. Yellow asterisks indicate vacuole-like compartments throughout their lifetime in the Dbn-/- time lapse video.

Title: Supplementary Movie 7

Description: Time-lapse video of GFP-RAB8A and microtubules at the leading edge of a Dbn-/- astrocyte before and after adding the Arp2/3 inhibitor CK-666, corresponding to Figure 4E. Video recording began 5 min before adding CK-666. The effect of CK-666 was followed for 5 min.
