# Supplementary material for: Drebrin controls scar formation and astrocyte reactivity upon traumatic brain injury by regulating membrane trafficking
Source: Nat Commun. 2021 Mar 5;12:1490. doi: 10.1038/s41467-021-21662-x (PMC7935889; doi:10.1038/s41467-021-21662-x)
Supplement: Supplementary file 11 — Reporting Summary [file 41467_2021_21662_MOESM11_ESM.pdf]

nature research

Corresponding author(s): Britta J. Eickholt
Last updated by author(s): Jan 24, 2021

# Reporting Summary

Nature Research wishes to improve the reproducibility of the work that we publish. This form provides structure for consistency and transparency in reporting. For further information on Nature Research policies, see our Editorial Policies and the Editorial Policy Checklist.

## Statistics

For all statistical analyses, confirm that the following items are present in the figure legend, table legend, main text, or Methods section.

| n/a | Confirmed | |
|---|---|---|
| ☐ | ☒ | The exact sample size (*n*) for each experimental group/condition, given as a discrete number and unit of measurement |
| ☐ | ☒ | A statement on whether measurements were taken from distinct samples or whether the same sample was measured repeatedly |
| ☐ | ☒ | The statistical test(s) used AND whether they are one- or two-sided<br>*Only common tests should be described solely by name; describe more complex techniques in the Methods section.* |
| ☒ | ☐ | A description of all covariates tested |
| ☐ | ☒ | A description of any assumptions or corrections, such as tests of normality and adjustment for multiple comparisons |
| ☐ | ☒ | A full description of the statistical parameters including central tendency (e.g. means) or other basic estimates (e.g. regression coefficient) AND variation (e.g. standard deviation) or associated estimates of uncertainty (e.g. confidence intervals) |
| ☐ | ☒ | For null hypothesis testing, the test statistic (e.g. *F*, *t*, *r*) with confidence intervals, effect sizes, degrees of freedom and *P* value noted<br>*Give P values as exact values whenever suitable.* |
| ☒ | ☐ | For Bayesian analysis, information on the choice of priors and Markov chain Monte Carlo settings |
| ☒ | ☐ | For hierarchical and complex designs, identification of the appropriate level for tests and full reporting of outcomes |
| ☒ | ☐ | Estimates of effect sizes (e.g. Cohen's *d*, Pearson's *r*), indicating how they were calculated |

*Our web collection on statistics for biologists contains articles on many of the points above.*

## Software and code

Policy information about availability of computer code

| | |
|---|---|
| Data collection | We customized a code to detect and quantify membrane tubules via FiJi/ImageJ. The explicit code is provided on Github: https://github.com/jschiweck/TubuleMacro.git |
| Data analysis | Graph pad version 7.0. Fiji ImageJ 1.51n. Imaris version 8.1.2 Inkscape 1.0.1 |

For manuscripts utilizing custom algorithms or software that are central to the research but not yet described in published literature, software must be made available to editors and reviewers. We strongly encourage code deposition in a community repository (e.g. GitHub). See the Nature Research guidelines for submitting code & software for further information.

## Data

Policy information about availability of data

All manuscripts must include a data availability statement. This statement should provide the following information, where applicable:

- Accession codes, unique identifiers, or web links for publicly available datasets
- A list of figures that have associated raw data
- A description of any restrictions on data availability

All data supporting the findings of this study are provided within the paper and its supplementary information. A source data file is provided with this paper. All additional information will be made available upon reasonable request to the authors. Cartoons and schemes in this manuscript were created by J.S and K.M with Biorender.com

# Field-specific reporting

Please select the one below that is the best fit for your research. If you are not sure, read the appropriate sections before making your selection.

☒ Life sciences ☐ Behavioural & social sciences ☐ Ecological, evolutionary & environmental sciences

For a reference copy of the document with all sections, see nature.com/documents/nr-reporting-summary-flat.pdf

# Life sciences study design

All studies must disclose on these points even when the disclosure is negative.

| | |
|---|---|
| Sample size | Sample sizes were not pre-calculated but chosen based on estimated effects in preliminary experiments as well as published literature. Given the magnitude of the effect of interest and thus resulting high statistical power, we considered a minimum of 3 animals/conditions sufficient. All cell biology experiments were performed using at least 3 independent biological repeats. The magnitude of the effects when using 3 independent samples provided statistical power to perform the necessary analyses. The individual in vivo experiments was conducted independently of each other. Quantitative live imaging analyses are based on 8-180 cells from at least 3 independent experiments. The exact numbers are defined in the appropriate figure legend. |
| Data exclusions | No data was excluded from any of the analyses reported in this study. |
| Replication | All experiments were performed using at least 3 independent biological repeats, while the mean ± standard error of the mean (SEM) was determined. All experiments shown were reproducible. |
| Randomization | Experimental groups were assembled such that controls and positive/negative experimental conditions were generated, processed and analysed in parallel. Samples and organisms were allocated to the respective group based on their genotype. Thus, sample randomization was not applicable to this study. Image acquisition was performed with randomly selected cells/fields. |
| Blinding | Blinding was not used during the study, as the cells or animals' identity was obvious by strong phenotypes on visible markers: The in vivo phenotype after stab wound injury in Dbn -/- mice were immediately apparent to the observer. Blinding was not used during live-cell imaging: The reduced number of Rab8+ tubular endosomes in Dbn -/- cells was immediately apparent to the observer. During analysis, the scientist was blinded to the genotype and drug treatment by allocating IDs to the cells rather than the description of the genotype, and a semi-automated macro with standard parameters for Rab8+ tubular endosome analysis was employed. Western blotting experiments were not blinded to ensure proper loading of the gel. Further experiments were not blinded to assure proper genotypes were used for the corresponding analyses. |

# Reporting for specific materials, systems and methods

We require information from authors about some types of materials, experimental systems and methods used in many studies. Here, indicate whether each material, system or method listed is relevant to your study. If you are not sure if a list item applies to your research, read the appropriate section before selecting a response.

## Materials & experimental systems

| n/a | Involved in the study | |
|---|---|---|
| ☐ | ☒ | Antibodies |
| ☐ | ☒ | Eukaryotic cell lines |
| ☒ | ☐ | Palaeontology and archaeology |
| ☐ | ☒ | Animals and other organisms |
| ☒ | ☐ | Human research participants |
| ☒ | ☐ | Clinical data |
| ☒ | ☐ | Dual use research of concern |

## Methods

| n/a | Involved in the study | |
|---|---|---|
| ☒ | ☐ | ChIP-seq |
| ☒ | ☐ | Flow cytometry |
| ☒ | ☐ | MRI-based neuroimaging |

## Antibodies

| | |
|---|---|
| Antibodies used | Antibodies and their used concentrations in Western blotting (WB), immunofluorescence (IF), and immunohistochemistry (IHC). Several lots of each antibody were used over the years: Mouse anti-DBN M2F6 (Enzo Lifesciences, ADI-NBA-110-E, IF & IHC 1:100; WB 1:1000), rabbit anti-GFAP (Synaptic Systems, 173 002, IF & IHC 1:1000), guinea pig anti-GFAP (Synaptic Systems, 173 004, IHC 1:400), guinea pig anti-S100β (Synaptic Systems, 287 003, IHC 1:200); rabbit anti-S100β (Atlas Antibodies, HPA015768, IHC 1:1000), mouse anti-alpha tubulin DM1a (Sigma Aldrich, T6199, WB 1:5000), mouse anti-NeuN A60 (Millipore, MAB377, IHC 1:1000), rabbit anti-IBA1 (Wako, 019-19741, IHC 1:500), anti-MAP2 (Synaptic systems, 188 004, IHC 1:500), mouse anti-GAPDH 6C5 (Abcam, ab8245, Wb 1:5000), mouse anti-RAB8 (BD Biosciences, 610844, WB 1:1000), goat anti-pan RAB8 (Sicgen, AB3176-200, IF & IHC 1:100), rabbit anti-integrin beta1 (Cell Signaling, #4706S, WB 1:1000), mouse anti-CD29 18/CD29 (BD Biosciences, 61046, WB 1:1000), rat anti-active integrin beta1/CD29 9EG7 (BD Pharmingen, 553715, IF 1:100), rabbit anti-integrin beta1 c-term (LSBio, LS-C413122, IF 1:100), rabbit anti-paxillin (Genetex, GTX125891, IF (1:250), rabbit anti-GST (Abcam, #9085-200µl, WB 1:500). Horseradish peroxidase (HRP)- |

conjugated secondary antibodies (WB 1:5000): Goat Anti-Rabbit IgG Antibody (H+L) (VectorLabs, PI-1000), Horse Anti-Mouse IgG Antibody (H+L) (Vectorlabs, PI-2000); Cross-absorbed secondary antibodies conjugated to cyanine or Alexa dyes were purchased from Dianova (IF & IHC 1:250): Donkey IgG anti-Mouse IgG (H+L)-Alexa Fluor 488 (Dianova, 715-545-150), Donkey IgG anti-Mouse IgG (H+L)-Alexa Fluor 647 (Dianova, 715-605-150); Donkey IgG anti-Goat IgG (H+L)-Alexa Fluor 488 (Dianova,705-545-147), Donkey IgG anti-Guinea Pig IgG (H+L)-Cy3 (Dianova,706-165-148), Donkey IgG anti-Rabbit IgG (H+L)-Alexa Fluor 647 (Dianova, 711-605-152), Donkey IgG anti-Rabbit IgG (H+L)-Alexa Fluor 488 (Dianova, 711-545-152), Donkey IgG anti-Rabbit IgG (H+L)-Cy3 (Dianova, 711-165-152).

Validation

Our laboratory previously validated the mouse anti-DBN M2F6 in DBN-/- and WT animals by Western blotting and immunohistochemistry (Wilmes et al., Sci Rep 2017). In this study, the specificity of the goat anti-pan Rab8 (used in immunocytochemistry+ immunohistochemistry) and mouse anti-RAB8 antibody (used for westernblotting) immunoreactivity was confirmed by analyzing Rab8a & Rab8b depleted astrocytes in immunocytochemistry (Figure 3A) and Western blotting (Figure S6C). Other antibodies are commonly used markers in immunocytochemistry and immunohistochemistry and produced the expected patterns, according to the manufacturers validation and previous publications. Antibodies used in Western blotting detected their targets at their expected and specific molecular sizes. For antibodies used in immunofluorescence, specificity was tested using positive and negative controls and checking their patterns with previously published results and manufacturer websites.
Validation for commerical antibodies can be found on the following websites:
Mouse anti-DBN M2F6 (Enzo Lifesciences, ADI-NBA-110-E): https://www.enzolifesciences.com/ADI-NBA-110/drebrin-monoclonal-antibody-m2f6/
rabbit anti-GFAP (Synaptic Systems, 173 002): https://www.sysy.com/product/173002
guinea pig anti-GFAP (Synaptic Systems, 173 004): https://www.sysy.com/product/173004
guinea pig anti-S100β (Synaptic Systems, 287 003): https://www.sysy.com/product/287003
rabbit anti-S100β (Atlas Antibodies, HPA015768): https://www.atlasantibodies.com/products/antibodies/primary-antibodies/triple-a-polyclonals/s100b-antibody-hpa015768/
mouse anti-alpha tubulin DM1a (Sigma Aldrich, T6199): https://www.sigmaaldrich.com/catalog/product/sigma/t6199
mouse anti-NeuN A60 (Millipore, MAB377): https://www.merckmillipore.com/DE/de/product/Anti-NeuN-Antibody-clone-A60,MM_NF-MAB377
rabbit anti-IBA1 (Wako, 019-19741): https://labchem-wako.fujifilm.com/us/category/01213.html
anti-MAP2 (Synaptic systems, 188 004): https://sysy.com/product/188004
mouse anti-GAPDH 6C5 (Abcam, ab8245): https://www.abcam.com/gapdh-antibody-6c5-loading-control-ab8245.html
mouse anti-RAB8 (BD Biosciences, 610844): https://www.bdbiosciences.com/us/reagents/research/antibodies-buffers/cell-biology-reagents/cell-biology-antibodies/purified-mouse-anti-rab8-4rab8/p/610844
goat anti-pan RAB8 (Sicgen, AB3176-200): http://www.sicgen.pt/product/rab8-polyclonal-antibody_1_19
rabbit anti-integrin beta1 (Cell Signaling, #4706S): https://www.cellsignal.com/products/primary-antibodies/integrin-b1-antibody/4706
mouse anti-CD29 18/CD29 (BD Biosciences, 61046): https://www.bdbiosciences.com/eu/applications/research/stem-cell-research/pluripotent-stem-cell-markers-esc-and-ipsc/human/purified-mouse-anti-cd29-18cd29/p/610467
rat anti-active integrin beta1/CD29 9EG7 (BD Pharmingen, 553715): https://www.bdbiosciences.com/us/applications/research/stem-cell-research/cancer-research/mouse/purified-rat-anti-mouse-cd29-9eg7/p/553715
rabbit anti-integrin beta1 c-term (LSBio, LS-C413122): https://www.lsbio.com/antibodies/itgb1-antibody-integrin-beta-1-antibody-cd29-antibody-c-terminus-icc-if-immunofluorescence-ihc-wb-western-ls-c413122/425557
rabbit anti-paxillin (Genetex, GTX125891): https://www.genetex.com/Product/Detail/Paxillin-antibody/GTX125891
rabbit anti-GST (Abcam, #9085-200µl): https://www.abcam.com/gst-antibody-ab9085.html
Goat Anti-Rabbit IgG Antibody (H+L), peroxidase (VectorLabs, PI-1000): https://vectorlabs.com/peroxidase-goat-anti-rabbit-igg-antibody.html
Horse Anti-Mouse IgG Antibody (H+L), peroxidase (VectorLabs, PI-2000-1): https://vectorlabs.com/peroxidase-horse-anti-mouse-igg-antibody.html
Donkey IgG anti-Mouse IgG (H+L)-Alexa Fluor 488 (Dianova, 715-545-150) https://www.dianova.com/shop/715-545-150-esel-igg-anti-maus-igg-hl-alexa-fluor-488-minx-bockgogphshohurbsh/
Donkey IgG anti-Mouse IgG (H+L)-Alexa Fluor 647 (Dianova, 715-605-150): https://www.dianova.com/shop/715-605-150-esel-igg-anti-maus-igg-hl-alexa-fluor-647-minx-bockgogphshohurbsh/
Donkey IgG anti-Goat IgG (H+L)-Alexa Fluor 488 (Dianova, 705-545-147): https://www.dianova.com/shop/705-545-147-esel-igg-anti-ziege-igg-hl-alexa-fluor-488-minx-ckgphshohumsrbrt/
Donkey IgG anti-Guinea Pig IgG (H+L)-Cy3 (Dianova, 706-165-148): https://www.dianova.com/shop/706-165-148-esel-igg-anti-meerschweinchen-igg-hl-cy3-minx-bockgohshohumsrbrtsh/
Donkey IgG anti-Rabbit IgG (H+L)-Alexa Fluor 647 (Dianova, 711-605-152): https://www.dianova.com/shop/711-605-152-esel-igg-anti-kaninchen-igg-hl-alexa-fluor-647-minx-bockgogphshohumsrtsh/
Donkey IgG anti-Rabbit IgG (H+L)-Alexa Fluor 488 (Dianova, 711-545-152):
https://www.dianova.com/shop/711-545-152-esel-igg-anti-kaninchen-igg-hl-alexa-fluor-488-minx-bockgogphshohumsrtsh/
Donkey IgG anti-Rabbit IgG (H+L)-Alexa Fluor Cy3 (Dianova, 711-165-152):
https://www.dianova.com/shop/711-165-152-esel-igg-anti-kaninchen-igg-hl-cy3-minx-bockgogphshohumsrtsh/

# Eukaryotic cell lines

Policy information about cell lines

| | |
|---|---|
| Cell line source(s) | HEK293TN (SBI/BioCat) LV900A-1-GVO-SBI |
| Authentication | HEK293TN cell line used was not authenticated. |
| Mycoplasma contamination | HEK293TN cell line tested negative for mycoplasma contamination |
| Commonly misidentified lines (See ICLAC register) | No commonly misidentified cell-lines were used in this study. |

## Animals and other organisms

Policy information about studies involving animals; ARRIVE guidelines recommended for reporting animal research

| | |
|---|---|
| Laboratory animals | Laboratory mouse strains: C57BL/6J (referred to as WT), female & male, ages: E16.5, P2, P30; B6.CMV:Cre/Dbnfl/fl (referred to as DBN-/-), female & male, ages: E16.5, P2, P30; B6.CAMK:Cre/Dbnfl/fl; female & male, age: P30; Tg(Aldh1l1-EGFP)OFC789Gsat/Mmucd (referred to as BAC Aldh1L1 eGFP mice), female & male, age: P30. Mice were housed in individually ventilated cages (IVCs). The cages contained wooden bedding material (SafeR Select, Safe, Augy, France), nestlets (Ancare, UK agents, Lillico, United King-dom), and a red, triangular plastic house (length: 12,5 cm, width: 11 cm, height: 6 cm; Tecniplast, Italy) or a plastic tunnel (length: 10 cm, diameter: 4,5 cm, in-house fabrication). The animals were maintained under standard conditions (room temperature: 22 ± 2 °C; relative humidity: 55 ± 10%) on a light:dark cycle of 12:12 h of artificial light (lights on from 6:00 a.m. to 6:00 p.m.). The mice were fed pelleted mouse diet ad libitum (V1534-000, Ssniff, Soest, Germany) and had free access to tap water at all times. |
| Wild animals | This study does not involve wild animals. |
| Field-collected samples | This study does not involve field-collected samples. |
| Ethics oversight | All animals were handled in accordance with the relevant national guidelines and regulations. Protocols were approved by the 'Landesamt für Gesundheit und Soziales' (LaGeSo; Regional Office for Health and Social Affairs) in Berlin, and animals are under the permit number G0189/14. |

Note that full information on the approval of the study protocol must also be provided in the manuscript.
